# The Impact of Selected *Lachancea* Yeast Strains on the Production Process, Chemical Composition and Aroma Profiles of Beers

**DOI:** 10.3390/molecules29235674

**Published:** 2024-11-30

**Authors:** Marek Zdaniewicz, Paweł Satora, Paulina Kania, Adam Florkiewicz

**Affiliations:** 1 Department of Fermentation Technology and Microbiology, Faculty of Food Technology, University of Agriculture in Krakow, Balicka Street 122, 30-149 Krakow, Poland; 2 Centre for Innovation and Research on Prohealthy and Safe Food, University of Agriculture in Krakow, Balicka Street 104, 30-149 Krakow, Poland; 3 Department of Food Analysis and Quality Assessment, Faculty of Food Technology, University of Agriculture in Krakow, Balicka Street 122, 30-149 Krakow, Poland

**Keywords:** beer, aroma, olfactometry, fermentation, yeast

## Abstract

Changing trends in the brewing market show that breweries want to attract consumers with new products. New flavours and aromas in beer can be achieved by using various additives. However, non-*Saccharomyces* yeast strains make it possible to produce beer with an original sensory profile but according to a traditional recipe (without additives). The aim of this study was to evaluate the influence of 10 different yeast strains, belonging to the species *Lachancea thermotolerans* and *L. fermentati*, on the creation of different physico-chemical profiles in beers. For this purpose, the same malt wort with a 12°P extract, hopped with Octawia hops (8.4% alpha acids), was inoculated with the aforementioned yeast strains. The fermentation kinetics, the yeast’s ability to ferment sugars, the production of organic acids and glycerol and the formation of volatile compounds in the beer were monitored. The beers obtained were classified as low-alcohol and regular. In addition, some beers were measured to have a low pH, qualifying them as “sour” beers, which are currently gaining in popularity. Most interesting, however, was the effect of the selected *Lachancea* yeast strains on the composition of the beer volatiles. In the second stage of this study, the beers obtained were again subjected to a chromatographic analysis, this time using an olfactometric detector (GC-O). This analysis was dictated by the need to verify the actual influence of the compounds determined (GC-MS) on the creation of the final aroma profile. This study showed that selected strains of *Lachancea thermotolerans* and *L. fermentati* have very high brewing potential to produce different original beers from the same hopped wort.

## 1. Introduction

Beer is one of the most widely consumed beverages in the world [1]. The popularity of this beverage is partly due to the variety of chemical compounds present in its composition [2]. These include those responsible for its refreshing effect [3], palate fullness/mouthfeel [4] and, most importantly, taste and aroma [5]. An analysis of the above attributes shows that the selection of an appropriate yeast strain is of paramount importance in influencing the characteristics in question [6]. Carbon dioxide, which is responsible for the refreshing effect of beer, is a major by-product of efficient yeast fermentation [7]. The flavour of beer is closely related to the yeast’s use of individual sugars and its ability to utilise nitrogenous compounds [8]. The organic acids produced by the yeast activity allow beers to be classified, for example, as sour beers (which are becoming increasingly popular) [9]. In addition, the glycerol synthesised by yeast cells is important to its so-called mouthfeel [10]. With regard to volatile compounds, yeasts can be used to create (by biosynthesis, biorelease or biotransformation) or reduce (by bioreduction or biotransformation) the concentration of key components of beer’s aroma [11]. In addition, the amount of alcohol produced by yeasts allows the resulting beers to be categorised into different groups, including non-alcoholic, low-alcohol, regular and strong beers [12]. The ethanol content has a significant influence on the energy value of the beverage [13]. This is particularly important in the context of evolving dietary preferences, where informed consumers are concerned not only with the flavour and aroma of a product but also with its alcohol content and caloric value [14]. It is noteworthy that the currently commercially available strains of *Saccharomyces cerevisiae* and *S. pasteurianus* yeast only represent a fraction of the total beer-producing yeast strains [15], and the full extent of their diversity remains largely unknown. Consequently, a significant amount of scientific research is devoted to the pursuit of novel strains from the non-*Saccharomyces* yeast group. Moreover, this research includes the use of single strains [16], as well as sequential fermentations [17,18]. The latter approach seems particularly promising in terms of the complexity of the final product profiles. However, it also poses certain technological challenges, including the reuse of yeasts.

In the present study, the authors chose to conduct a series of fermentation experiments with the aim of selecting *Lachancea* yeast strains with high potential for the production of different types of beer. The decision to test the aforementioned yeasts was made due to their prevalence in the production of wine and other beverages (such as mead) [19]. Some display high tolerance to alcohol concentration, while others show the ability to produce significant amounts of acids and glycerol [20,21]. In addition, some show remarkable resistance to osmotic pressure [22]. Our previous studies have identified a strain that displays resistance to hop compounds while exhibiting minimal organic acid synthesis. Accordingly, the present study was designed to include a wider range of yeast strains, specifically *L. fermentati* and *L. thermotolerans*. Some of these additional strains were selected for their potential to ferment maltose, a primary sugar present in malt wort, as reported by Madden et al. [23]. Furthermore, the analytical part was complemented by the results of an olfactometric analysis (GC-O). As a result, the beers obtained could be classified in terms of the potential of the yeast to produce different beers (sour, low-alcohol or regular) and subjected to a detailed chemical analysis using both MS-TOF and the olfactometric detector. This made it possible to demonstrate the range of changes in the concentrations of the individual beer volatiles depending on the strain used and to identify the dominant aromas and the compounds responsible for them.

## 2. Results

### 2.1. The Influence of Selected Lachancea Yeast Strains on the Kinetics of Beer Fermentation

Cold wort with 12% *w*/*w* extract was inoculated with *L. thermotolerans* (MN477031, PYCC6986, PYCC7194, PYCC2908, PYCC4135, PYCC4675, PYCC5195 and PYCC6375) and *L. fermentati* (PYCC5883 and PYCC6701) yeast strains. The intensity of the process was measured in a manner analogous to the method of Siesto et al. [24], using the amount of CO_2_ evolved as a metric. The quantity of gas released reflected the utilisation of sugars by the yeast strains studied for their production of alcohol and other by-products. This can be seen as an indirect reflection of their fermentative capabilities [25].

**Figure 1:**
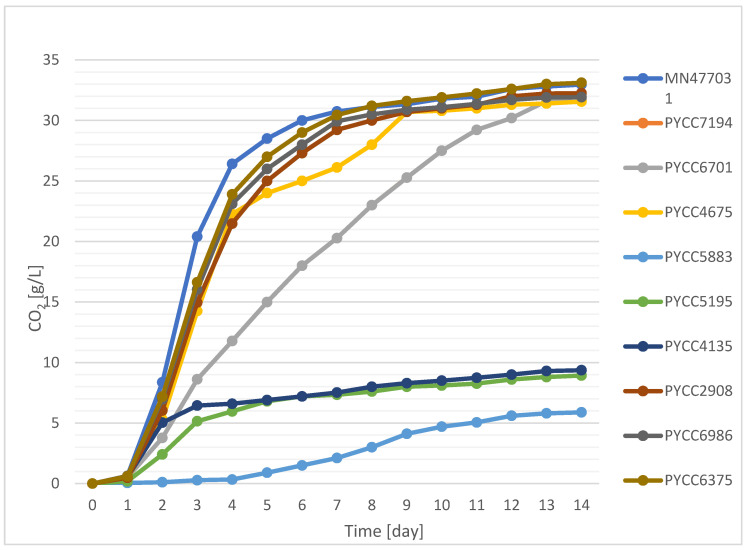
Kinetics of malt wort fermentation process using selected *Lachancea* strains.

On the first day of fermentation (Figure 1), a modest, uniform release of CO_2_ was observed in all samples. This was due to the need for the yeast to adapt to the new environment during the so-called lag phase. This phase is frequently observed in the fermentation process, including that of grape musts, despite the presence of a considerable amount of monosaccharides, which are mainly used by the yeast [26,27]. In experiments carried out by Fairbairn et al. [28], in which *S. cerevisiae* monocultures were inoculated and sequential fermentations of *L. thermotolerans* and *S. cerevisiae* were carried out, it was shown that the lag phase lasted approximately 24 h regardless of the supplementation of nitrogenous compounds in the wort, a finding that is consistent with the results of our study. In addition, the duration of the lag phase did not vary significantly between the strains, with the exception of PYCC5883. The intensity of the process was influenced by the amount of CO_2_ released from the second day of fermentation onwards. It is noteworthy that these differences were also observed between strains of the same species of *L. fermentati* and *L. thermotolerans*. Consequently, each strain was evaluated individually, as in the study by du Plessis [29]. On the second day of the process, the highest amount of carbon dioxide released among the samples was observed in the case of strain MN477031 (8.37 g/L), while the lowest result was recorded for *L. fermentati* strain PYCC5883 (0.11 g/L). It is noteworthy that the differences between the yeast strains became more pronounced as the fermentation progressed. As a result, the samples could be divided into at least two distinct groups. One of these was PYCC5883, which exhibited a prolonged time to start fermentation that lasted until the fourth day of the process. However, this was followed by a rapid increase in the fermentation intensity, culminating in a final weight loss of 5.89 g/L, which was most similar to that of samples PYCC5195 and PYCC4135 (8.93–9.37 g/L). Such long times for the process to start are typically observed in the fermentation of highly concentrated media such as mead [30] but are unusual in brewing. In some cases, the fermentation process started almost immediately, with a short lag phase. In the study by Zdaniewicz et al. [31], a decrease in the apparent extract of about 1% (*w*/*w*) was observed as early as on the first day of fermentation in industrial beer production trials. In the study by Sampaolesi et al. [32], the term “lager behaviour” was used to describe a brewing yeast that exhibited a prolonged lag phase, a low fermentation intensity and a significant release of carbon dioxide at the end of the process. In the case of strain PYCC5883, the prolonged time for it to start the process may have been caused by its poor fermentation properties and inability to ferment maltose, maltotriose and similar polysaccharides. Moreover, the amount of CO_2_ evolved at the end of the process did not exceed that observed in the other samples. Therefore, despite the longer time for it to start the process, this cannot be considered typical “lager behavior”. In the intensive fermentation group, we can see, among others, strain MN477031, which had the highest CO_2_ secretion by up to the 7th day of the process (30.74 g/L), and strain PYCC6375, which had similar characteristics to the previous strain (30.44 g/L). It is noteworthy that the decrease in the final mass of these samples, related to the release of CO_2_, was more than three times higher than that of the previously mentioned strains from the low-process-intensity group (PYCC5195 and PYCC4135). The next strains in terms of gas release were PYCC2908 (29.22 g/L) and PYCC5195 (29.93 g/L). It is also noteworthy that the values of evolved CO_2_ from days 7 to 14 showed considerable strain-dependent variation. Some strains reached a near-final value and subsequently released only minimal additional amounts of gas, whereas others (e.g., PYCC6701) showed sustained process intensity. Previous studies have shown that the process intensities can vary depending on the strain and fermentation medium used. In the study by Zdaniewicz et al. [16], an initially intense fermentation process was observed for strain MN477031, even more so than that for *S. cerevisiae*. This was observed independently of the IBU value of the wort. However, as observed in the present study, this intensity decreased significantly from day 7 of the process. These results suggest that both *L. fermentati* and *L. thermotolerans* strains have considerable potential for the fermentation of hopped malt wort. However, the intensity and extent of the fermentation process are strain-specific characteristics. Although Vicente et al. [25] found similarities in the fermentation kinetics of the *L. thermotolerans* strains tested during the fermentation of grape must, they also identified at least one strain with significantly inferior fermentation properties compared to those of the others (a weight loss of 2.0% vs. 2.5%). In a brewing trial, Postigo et al. [9] tested selected *L. thermotolerans* strains for their ability to produce sour beers at different scales (from 100 mL to 100 L). At the smaller scale, the values obtained were similar to those of our strains (which showed the highest fermentability), but the process was completed much faster (by day 5). The main reason for this discrepancy was the use of mechanical sample mixing at the smaller scale (100 mL), which significantly improved the process kinetics. At the 100 L scale, the process took approximately 20 days, which was significantly longer than the duration observed in our study. Interestingly, almost from the smallest scale, all the strains studied showed a similar fermentation capacity. Given the considerable discrepancy in the kinetics observed in our study, we proceeded to assess the potential of each strain for beer production based on the subsequent analysis of the acid, alcohol and volatile compound contents of the beers produced with their participation.

### 2.2. The Effect of Selected Lachancea Yeast Strains on the Concentration of Sugars, Glycerol and Real Extract in Beers

The CO_2_ produced during fermentation (Figure 1) showed a strong correlation with the yeast’s ability to utilise different wort sugars. The sequence of sugar utilisation by the yeast *S. cerevisiae* has been extensively studied by many authors. It has been shown that glucose and fructose are utilised first, followed by maltose and maltotriose [27,33]. The final concentration of sugars in the beer was determined for the yeast strains studied. From these analyses, it was possible to determine the ability of the strains to use specific sugars as a carbon source. In the study by Madden et al. [23], strains of *L. thermotolerans* (CPD-28-3, CPD-10-1, CPD-12-1, CPD-05-1, CPD-04-1, CPD-09-1, CPD-27-1 and CPD-06-1) and *L. fermentati* (VS93A and VS92A) showed the ability to utilise maltose. Given that maltose is the primary sugar in malt wort, it was decided that it would be worthwhile to test other strains of *L. thermotolerans* and *L. fermentati* in terms of beer production. As documented in the literature, maltose is the main sugar component in wort, accounting for approximately 60% of its total sugar [34]. In our study (Table 1), the content of maltose in wort with an extract of 12% *w*/*w* was determined to be around 58 g/L, which is an intermediate result compared to those of other studies: around 50 g/L [24,35] and 75 g/L [36]. The ability of the yeast to utilise the sugar showed considerable variation between strains, with rates ranging from 0% to over 90% in our study. A comparable range of sugar utilisation was observed by Toh et al. [35].

Strains MN477031, PYCC4675 and PYCC6986 had the highest primary sugar utilisation, with approximately 4–6 g/L remaining in the beer. In contrast, strains PYCC5883, PYCC5195 and PYCC4135 had the lowest sugar consumption, with no significant change in the wort content (53–58 g/L). The remaining strains showed intermediate values, ranging from about 8 to 17 g/L, which are relatively high compared to the results of previous studies: 2.75 g/L [36] and 0.08 g/L [24]. Ranking yeast strains in terms of their maltose utilisation is a valuable tool for assessing the potential of a strain to produce beers with a regular alcohol content. Therefore, strains that may have been used to produce a significant amount of alcohol in the context of winemaking (with a high concentration of simple sugars) may prove to be much less advantageous in the context of brewing (with a high concentration of maltose). This is due to the different sources of carbon available in the environment. To achieve comparable results, it would be advisable to use exogenous enzymes to increase the glucose concentration in the solution [37]. However, this initial disadvantage could potentially become an advantage for the production of beverages with a reduced alcohol content. This approach eliminates the need to interrupt the process by rapidly cooling the tank’s contents and/or using lower amounts of wort extract.

Subsequently, the ability of the strains to utilise simple sugars, in addition to sucrose, was assessed through further investigations. In the study by Galaz and Franco [36], glucose was completely utilised by yeast isolated from insects. In our study, the concentration of glucose in the final product ranged from about 0.7 g/L to about 3.6 g/L depending on the strain used. In experiments conducted by Postigo et al. [9], it was observed that the residual sugar content in beer can vary significantly depending on the measurement scale used. The glucose concentrations were even reported to be almost twice as high in 1 L samples (0.10 g/L) compared to 100 L fermentations (0.19 g/L). In the case of synthetic musts, a study by Vicente et al. [25] showed that *L. thermotolerans* strains used between 52 and 72% of the simple sugars. Even the *S. cerevisiae* strain left approximately 17 g/L of glucose and fructose combined in the product, a level even higher than that observed in the pre-fermentation wort.

The initial sucrose concentration was approximately 4 g/L, which is consistent with data from the literature for 12% *w*/*w* wort [38]. Upon examination of the final beer product, the majority of the strains were found to have fully utilised the aforementioned sugars. However, there were a few exceptions, namely strains PYCC5883, PYCC5195 and PYCC4135, which showed a residual sugar content of approximately 3 g/L. This observation is similar to that for maltose and indicates very low utilisation of this sugar.

With regard to the samples belonging to the most fermentable group (Figure 1), it is evident that the reduction in the extract value was the most pronounced. For beer MN477031, the real extract was approximately 5.82%, corresponding to a real degree of fermentation of 53%. The considerable variability in the results for the real extract and alcohol is indicative of the considerable diversity of the characteristics exhibited by the strains selected for study. In studies conducted by Domizo et al. [39] and Postigo et al. [9], the residual sugar levels in the beer samples were significantly lower (approximately 15–20 g/L) compared to our residual extract levels (ranging from 60 to 106 g/L). In addition, the majority of the selected strains exhibited high fermentability, making them unsuitable for the production of beers with a reduced alcohol content.

Glycerol is one of the additional metabolites produced during the fermentation process [40]. In beers, glycerol has favourable impacts on “mouthfeel” and other similar taste attributes (“smoothness”, “roundness”, etc.) [41]. According to Sohrabvandi et al. [42], the addition of glycerol at a dose of 0.3% to 2% improves the body and fullness of non-alcoholic beers. However, for regular beers, the positive concentration (range) of glycerol remains still unclear [41]. In our study, the amount of glycerol formed showed an inverse correlation with the amount of CO_2_ released during the process. This evidence supports the assertion that the fermentation process is indispensable to its formation. In the study by Vicente et al. [25] on winemaking with *L. thermotolerans* strains, glycerol was formed at amounts ranging from 2.49 g/L to 3.36 g/L, illustrating a variation of approximately 26% between samples. In addition, the authors noted that the discrepancy in the results regarding the effect of strain on the production of this compound may have been due to the use of mixed fermentations with *S. cerevisiae* strains, where identical conditions were not consistently maintained as they are in pure cultures. The results showed a greater degree of variation between strains, regardless of whether they belonged to the *L. thermotolerans* or *L. fermentati* group. In the samples fermented with MN477031 and PYCC6701, values above 6 g/L were recorded. On the other hand, the remaining samples showed values between 1.59 and 3.91 g/L, in agreement with the results of other researchers [9,25] and slightly higher than those reported by Domizio et al. [39].

### 2.3. The Effect of Selected Lachancea Yeast Strains on the Ethanol Concentration, pH and Caloric Value of the Beers Produced

Beer classification may be based on a number of factors. Some of these are the raw materials used in production (e.g., wheat beers), the type of fermentation used (bottom or top), colour (light or dark) and other physico-chemical parameters [43]. In this study, the strains were evaluated on the basis of their alcohol content, pH and calorific value (Table 2). It is noteworthy that the samples showed considerable variation in their ethanol production. Strains MN477031, PYCC4675 and PYCC6986 produced ethanol concentrations in excess of 4% (*v*/*v*), a result comparable to that observed in studies by Postigo et al. of both 1 L and 100 L scale fermentations. Interestingly, the scale used had no discernible effect on the amount of alcohol produced [9]. This offers significant potential for scaling up production based on the laboratory results. However, the results obtained were lower than those reported by Domizio et al. [39], who obtained an alcohol concentration of over 5% (*v*/*v*) from wort with an extract of 12.75° Plato. It is also noteworthy that samples with a similar low alcohol content (PYCC5893 and PYCC4135) and a comparable pH (Table 2) showed significant differences in their fermentation kinetics (Figure 1), lactic acid concentration (Table 3) and glycerol production (Table 1), among other variables. It is also important to consider the correlation between pH and lactic acid formation, as the influence of lactic acid on pH is a key factor in understanding the value of this parameter. The beers with the highest lactic acid formation were found to have the lowest pH, regardless of the amount of other acids present. The calorific value of the resulting beers was similar, at around 42–43 kcal/100 mL; however, the lower alcohol content of the PYCC5883 sample did not reduce its calorific value as much as expected.

### 2.4. The Effect of Selected Lachancea Yeast Strains on the Concentration of Organic Acids in Beers

Non-*Saccharomyces* yeasts are often recognised for their ability to produce organic acids, which appears to be a critical factor in the production of sour beers. The production of sour beers has primarily been achieved through the process of spontaneous fermentation, which results in the formation of organic acids that influence the flavour and low pH of the beer. In addition, lactic acid bacteria (LAB) and, more recently and increasingly popularly, lactic-acid-producing yeast starters [36] can be used in this process. From a technological point of view, the use of yeast strains that use sugar metabolism to produce alcohol and acids represents a notable advance. This technology is referred to as the LAB-free method [44].

In our study, we compared the ability of yeast strains to alter the levels of key organic acids. To this end, Table 3 summarises the content of each acid in the wort in relation to their levels in the finished beers produced using different *Lachancea* strains.

**Table 3:** Concentration of organic acids, glycerol and real extract in wort and beers fermented by the strains *L. thermotolerans* and *L. fermentati*.

	Citric Acid	Malic Acid	Succinic Acid	Lactic Acid	Acetic Acid
	g/L				
Wort	0.86 ± 0.12 ^ab^	2.58 ± 0.41 ^a^	0.53 ± 0.41	n/d	n/d
MN477031	0.68 ± 0.09 ^bcd^	1.97 ± 0.25 ^abc^	0.50 ± 0.47	0.01 ± 0.01 ^a^	0.3 ± 0.14 ^ac^
PYCC7194	0.78 ± 0.08 ^bcd^	2.14 ± 0.18 ^a^	0.51 ± 0.40	6.97 ± 0.55 ^b^	0.03 ± 0.02 ^b^
PYCC6701	0.58 ± 0.12 ^c^	1.74 ± 0.04 ^b^	0.45 ± 0.38	2.53 ± 0.81 ^c^	n/d
PYCC4675	0.72 ± 0.01 ^d^	1.87 ± 0.05 ^c^	0.46 ± 0.28	1.69 ± 0.11 ^c^	0.04 ± 0.12 ^abc^
PYCC5883	0.96 ± 0.11 ^ab^	2.40 ± 0.41 ^a^	0.57 ± 0.48	0.83 ± 0.05 ^d^	2.83 ± 0.57 ^d^
PYCC5195	0.82 ± 0.22 ^abcd^	2.22 ± 0.23 ^a^	0.52 ± 0.18	0.12 ± 0.05 ^e^	0.53 ± 0.41 ^bc^
PYCC4135	0.78 ± 0.11 ^abcd^	2.23 ± 0.21 ^a^	0.59 ± 0.76	0.13 ± 0.02 ^e^	0.16 ± 0.08 ^c^
PYCC2908	0.58 ± 0.06 ^c^	1.68 ± 0.14 ^bc^	0.41 ± 0.25	2.22 ± 0.09 ^c^	n/d
PYCC6986	0.64 ± 0.03 ^c^	1.76 ± 0.06 ^bc^	0.48 ± 0.35	0.68 ± 0.08 ^d^	0.01 ± 0.01 ^b^
PYCC6375	0.76 ± 0.04 ^abd^	2.22 ± 0.07 ^a^	0.53 ± 0.33	2.19 ± 0.05 ^c^	0.22 ± 0.10 ^ac^

Notes: The results are presented as the average of three or more independent replicate experiments. The standard deviation follows the ± symbol. Values with different superscript roman letters (a–e) in the same column are significantly different according to Tukey’s range test (*p* < 0.05).

The results obtained (Table 3) show that lactic and acetic acids were not present in the wort. Their concentration was a consequence of the fermentation process. Similarly, no lactic acid was detected in the wort of Galaz and Franco [36], and in the samples of Toh et al. [35], the level of acid was 1.12 g/L, which can be considered a high level. With regard to lactic acid, our study showed significant differences depending on the strain. The range in the compounds studied showed considerable variability, from 0.01 g/L to about 7 g/L. The highest lactic-acid-producing ability was observed in strain PYCC7194, while the lowest was observed in MN477031, at 0.01 g/L. This finding is consistent with previous observations reported by Zdaniewicz et al. [16]. The remaining strains produced the aforementioned acid in amounts ranging from about 0.1 g/L to about 2 g/L, similar to the values obtained by Postigo et al. [9] and significantly higher values than those reported by Domizo et al. [39]. Similarly, the winery study by Vicente et al. [25] showed that the amount of acid produced was strain-specific. These results were comparable, with the exception of strains that produced less than 0.55 g/L and one strain that produced more than 5.18 g/L (6.97 g/L by PYCC7194).

The biosynthesis of acetic acid was found to be similar to that of lactic acid. Some strains produced minimal amounts of the compound (PYCC2908 and PYCC2908), while others showed relatively high production levels (PYCC5883). It is noteworthy that strain PYCC5883, for which it took the longest time for the fermentation to start (up to 4 days, Figure 1), produced almost 3 g/L of acetic acid. An explanation for this phenomenon may be the fact that acetic acid is produced by yeast in larger amounts as a result of stress factors in the environment; these may also be, among other things, a small amount of assimilable nutrients. In the case of strain PYCC2908, no correlation was observed between the acid synthesis and process kinetics. Interestingly, the results obtained for the other samples were similar to those reported in the study by Vicente et al. [25].

Another group of organic acids is that initially present in the wort, including malic acid (2.58 g/L), succinic acid (0.53 g/L) and citric acid (0.86 g/L). As a result of the fermentation process, the concentration of the first acid decreased in almost all cases. The reduction in the initial malic acid content ranged from about 0.2 g/L to about 1 g/L, which was lower than that in the samples of Vicente et al. [25], where the malic acid utilisation ranged from 16% to 54%. In the fermentation samples of Toh et al. [35], a reduction in the acid under investigation of around 60% was also observed, and this reduction was not dependent on the strain used. With regard to succinic acid, the changes in its concentration were not statistically significant. In winemaking, this acid can impart a salty (sometimes bitter) taste at low concentrations, while at higher concentrations (above 500 mg/L), it is described as harsh, very dry or even unnatural [45]. Despite the initial concentration in the wort being around 0.5 g/L and the yeast tested not showing any reduction, this subject has not been extensively researched in the brewing industry. In a study conducted by Toh et al. [35], a high concentration of the aforementioned acid was found, ranging from 1.76 to 2.63 g/L, depending on the yeast strain used. It is noteworthy that this acid was also present in high concentrations in the wort (4.11 g/L). Even higher concentrations of this acid in the resulting beers were recorded by Siesto et al. [24] (over 7 g/L), while Tyrell [46] reported the presence of this acid in beer to be at around 35–90 ppm. In the case of citric acid, its concentration in the wort was ca. 0.9 g/L, which was similar to the value obtained by Li and Liu (2015) (ca. 0.1 g/L) [47]. In our research, citric acid showed a slight decrease in concentration as a result of the fermentation process or no significant change. For strains PYCC2908 and PYCC6701, the highest level of utilisation was observed at around 30% relative to the wort. Liu and Li (2015) showed that the concentration of this acid in beer depends on its initial contents in the wort, and the fermentation process has a negligible effect on its final concentration, which was close to our findings [47].

### 2.5. The Effect of Selected Lachancea Yeast Strains on the Volatile Composition of the Beers Produced

The main objective of using non-*Saccharomyces* yeasts in beer production is to capitalise on their secondary metabolism, which facilitates the synthesis of flavour-active compounds during fermentation [17]. It is noteworthy that despite the large number of compounds present in beer (more than 800), only a few exert a significant influence on the sensory quality of the product [48]. The most important groups of volatile compounds in beer are esters and higher alcohols [49]. The fermentation trials showed considerable variation in the content of these volatile compounds depending on the strain used (Table 4). In many cases, the differences were remarkable not only between the *L. thermotolerans* and *L. fermentati* yeast types but also between individual strains.

Among the higher alcohols, isoamyl alcohol, isobutyl alcohol and phenylethanol are of particular interest due to their pronounced influence on the sensory profile of beer [50]. In the case of the former, its concentration was the highest compared to those of the other higher alcohols, which is in line with the results of previous studies [48]. It is characterised by an aroma reminiscent of balsamic alcohol in beer [32], and the threshold concentration (250 µg/L) was reached in all samples. It is also noteworthy that the type of yeast and the fermentation yield had no significant effect on the observed value. The highest concentration was observed in the PYCC6701 sample, while the lowest amount was produced by the PYCC5883 yeast. The study by Galaz and Franco [36] showed that *L. thermotolerans* strains had a significantly reduced ability to produce this compound compared to *L. quebecensis* yeast. Similarly, Toh et al. [35] showed that even *T. delbrueckii* Biodiva, which produces less than half the amount of ethanol, has a higher rate of isoamyl alcohol synthesis compared to *L. thermotolerans* yeast. This is an important finding, as isoamyl alcohol, when present in higher concentrations, imparts a more robust aroma in beer, which can affect its drinkability [48]. Isobutyl alcohol is considered an unfavourable component in beer when it is present at higher concentrations, especially when its concentration exceeds 20% of the sum of n-propanol, isobutyl alcohol and isoamyl alcohol [49]. The results of these experiments indicate that the content of the higher alcohol mentioned above is not significantly correlated with the type of yeast. Instead, significant differences were observed at the strain level. With regard to phenylethanol, it is evident that the amount produced was determined by the extent of the process. The strains that showed minimal CO_2_ release during fermentation (Figure 1) showed the lowest levels of phenyl ethanol production, ranging from 55 to 814 µg/L. In contrast, the amounts of phenylethanol were significantly higher for efficient fermentations (up to about 1700 µg/L for MN47708). It is worth noting that this compound is often considered to be an important component of the volatile profile of beer, despite its typical occurrence below the sensory threshold [49]. Other notable higher alcohols include 2,3-butanediol, which, like acetoin (3-hydroxy-2-butanone), results from the reduction of diacetyl (2,3-butanedione) [51]. Unlike diacetyl, it has a markedly high detection threshold, making it relatively easy to remain undetected in beer. In the present study, the compound was detected at very low levels, occurring only in the two samples (PYCC6701 and PYCC6986) with the highest alcohol concentrations. The presence of 2-methyl-3-buten-2-ol was detected in the majority of the samples, with its concentration showing a dependence not only on the specific yeast strain used but also on the incorporation of hops during the beer production process, as previously documented in the literature. This is due to its formation via the B-acid reaction during boiling [52]. However, the concentrations of this compound were relatively low in all samples, especially in comparison with those of isoamyl alcohol.

Acetate esters are considered to be the main group of esters present in beer. These include ethyl acetate (a solvent-like aroma), isoamyl acetate (a banana aroma) and phenylethyl acetate (a combination of rose and honey aromas). The second group of esters consists of ethyl esters, which include ethyl hexanoate (aniseed, apple-like aroma), ethyl acetate (sour apple aroma) and ethyl decanoate (floral aroma) [48,53]. In general, the beers with optimal fermentation yielded significantly higher concentrations of ester compounds compared to those in the beers where the fermentation process was less efficient. This phenomenon was particularly evident in the case of ethyl acetate, which was present at concentrations above 2000 µg/L in the samples with efficient fermentation, in contrast to the samples with a lower fermentation intensity (e.g., in PYCC5883 beer), where it was detected at 39 µg/L. The concentration of this compound was the subject of a comparative study between *L. thermotolerans* and *L. quebecensis* strains carried out by Galaz and Franco [36]. These authors showed that the *L. thermotolerans* strain was able to produce a significantly greater amount of ethanol while obtaining concentrations of ethyl acetate that were more than four times lower than those produced by the *L. quebecensis* strain. It is worth noting that the concentration found in our study was even lower, at around 2.6 ppm, compared to that in the above study, where it was around 7.9 ppm. Other volatile compounds were also identified at lower concentrations. The closest value was that of isoamyl acetate, which showed a difference of only 0.04 mg/L between strain PYCC 6701 and the *L. thermotolerans* isolate obtained from insects by Galaz and Franco [36]. A similar observation of the dependence of concentration on the process intensity may be applicable to ethyl acetate and ethyl hexanoate. Ethyl hexanoate is considered to be a crucial component of beer’s aroma due to its low sensory threshold (3 µg/L), which was reached in almost all of the experiments. The exceptions were samples PYCC5195 (with a very low fermentation yield) and PYCC7194, where its concentration was not recorded or was below the sensory threshold (PYCC5883—very low fermentation yield).

### 2.6. Potential of the L. thermotolerans and L. fermentati Strains for the Production of Different Types of Beers

**Figure 2:**
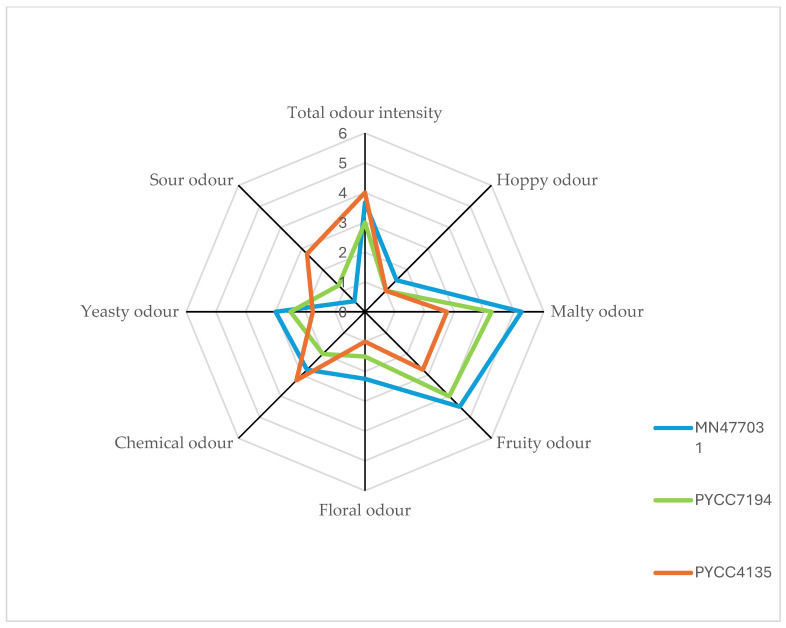
Sensory analysis (QDA) of beers produced with the use of selected *Lachancea* yeast strains. The intensity is marked from 0 to 6, where 0 is no detection while 6 is very high intensity.

The final part of this research was to demonstrate the potential of the strains analysed to produce different types of beer from the same wort. For this purpose, a two-stage selection of microorganisms was carried out. The first stage was based on the results of physico-chemical analyses. Taking into account the alcohol concentration, pH, amount of organic acids produced, fermentation capacity and glycerol content, the strains were divided into three groups (low-alcohol, sour and regular). Unfortunately, no strain could be recommended for the production of diet beers due to the small differences in the calorific value of the products. The first group of low-alcohol beers consisted of the following: strains PYCC5883, PYCC5195 and PYCC4135. The second group included the strains PYCC7194, PYCC6701 and PYCC2908. The remaining strains (MN477031, PYCC4675, PYCC6986 and PYCC6375) were considered useful for the production of regular beers. However, taking into account the results of the pH analysis (depending on the amount of acids), only strains MN477031 and PYCC6986 could be recommended for this group. In the second stage of strain selection, the number of recommended yeasts was reduced to one strain for each type of beer. This selection was based on the QDA. The results of the QDA for the strains with the highest potential are shown in Figure 2. On this basis, it can be seen that all of the beers were characterised by a moderate overall aroma (around 3–4 points). This is undoubtedly an advantage in the case of the regular beer, which mainly had noticeable malt and fruit aromas. The panel found these aromas desirable and well balanced. In addition, in the case of sour beers, it is worth noting the significantly lower level of hop aroma and the increased level of fruit aromas, which, according to the Beer Judge Certification Programme (BJCP), is a characteristic feature of sour beers [54]. In relation to the low-alcohol beer, a lower concentration of aromas formed during fermentation (fruity, floral) and a significantly higher level of chemical aromas can be observed, which influences the overall assessment of the beer’s aroma intensity. However, it should be noted that almost all of the aromas are moderate and therefore do not have a negative impact on the consumer perception of the beer produced.

A GC-O analysis was also carried out to determine which of the identified (GC-MS) compounds were actually perceived in the complex profile of the resulting beer (Table 5). This is particularly important, as in certain studies of beers, an olfactometric analysis was the only method capable of identifying the specific compounds responsible for the hop aroma of beer.

In fact, these compounds were different from those commonly reported in the literature [55]. The “regular” beer sample showed a higher concentration of aroma compounds than those in the sour and low-alcohol beer. In addition, the chromatographic analysis revealed a large number of compounds responsible for the aroma of the beers that do not belong to the higher alcohols and esters previously discussed. Examples of these compounds are dihydromyrcenol and β-damascenone. Dihydromyrcenol is a monoterpene alcohol [56] and is responsible for the citrus and herbal aromas of essential oils and redcurrants [57]. This compound was first identified in wines in 2017 by Alegre et al. [58] in a study using an olfactometric analysis of wines. Olfactometric analyses of beers have shown that β-damascenone is a significant component of wheat beers [59] and pilsner beers [60] due to its low sensory threshold. It is noteworthy that Schieberle [61] previously indicated that β-damascenone has the highest odour-active value for pale lagers. It is followed by other compounds such as ethyl butanoate, 3-methylbutanol, ethyl hexanoate and 2-phenylethanol, which have been identified as major aroma components [62]. In wines, it produces a sweeter, fruity character, in combination with isoamyl acetate [58]. In our study, it was described as sweet, candy-like or plummy, depending on the beer. This is similar to the observations of Tokita et al. [62], who described it as floral, honey-like or fruity.

## 3. Materials and Methods

### 3.1. Materials

#### 3.1.1. Yeast Strains

The *L. thermotolerans* strain MN477031, isolated from grape must in Slovakia, was used for fermentation. It was previously identified and characterised by sequencing of the 5.8S-ITS rRNA gene region (GenBank NCBI database accession number MN477031). The *L. thermotolerans* strains PYCC6986, PYCC7194, PYCC2908, PYCC4135, PYCC4675, PYCC5195 and PYCC6375 and the *L. fermentati* strains PYCC5883 and PYCC6701 were obtained from the Portuguese Yeast Culture Collection (PYCC), Caparica, Portugal.

#### 3.1.2. Hops

The Polish hop variety Oktawia, 8.4% alpha acids (Polish Hops, Karczmiska Pierwsze, Poland), was used.

### 3.2. Methods

#### 3.2.1. Wort Production

Industrial wort was produced from 1200 kg of pilsner malt (Ireks, Kulmbach, Germany) and 46 hL of water. The all-malt wort preparation was carried out according to the following mashing protocol (178 min): mashing in at 45 °C (10 min); heating to 52 °C (0.7 °C/min); rest (10 min); heating to 63 °C (1.38 °C/min); rest (25 min); transfer of the thick mash (15 hL, 5 min); heating to 72 °C (0.9 °C/min); rest (10 min); heating to 98 °C (1.04 °C/min); boiling the mash (10 min); returning the mash (10 min); rest at 70 °C (30 min); heating to 80 °C (1 °C/min); and rest at 80 °C (5 min). The wort was separated from the spent grains using a lauter tun. After lautering, 5 L of the wort was boiled for one hour under laboratory conditions. Oktawia hops (8.4% alpha acids) were added at 0.5 g/L at the start of boiling. After boiling, the hot trub was removed from the wort, and the samples were cooled to 22 °C and standardised to 12% extract with distilled water. Before inoculation, the main quality parameters of the wort were analysed.

#### 3.2.2. Yeast Propagation and Fermentation

All of the yeast strains were maintained on Sabouraud Dextrose LAB-AGAR (Biocorp, Poland) at 5 °C. Yeasts grown for 24 h on Sabouraud Dextrose LAB-AGAR at 25 °C were transferred into a flask containing 20 mL of liquid Sabouraud broth (Biocorp, Warszawa, Poland) and grown for 2 days at 25 °C in a rotary shaker shaken at 121 rpm. The yeasts were then centrifuged (735× *g*) for 15 min, washed with sterile distilled water, centrifuged again and suspended in 10 mL of hopped wort. The number of cells in 1 mL of suspension was assessed using a Thoma chamber (in triplicate).

A total of 200 g of hopped wort was inoculated with the *L. thermotolerans* or *L. fermentati* yeast cell suspensions to give 7.65 × 10^6^ cells/mL and fermented in rubber-stoppered Erlenmeyer flasks with fermentation tubes. The samples were fermented at 22 °C in a Q-CELL thermostatic chamber, with daily weight loss measurements. A weight loss below 0.2 g/day being obtained indicated the end of fermentation and prompted the start of cold conditioning of the bottled beers for at least 3 days at a temperature of 0 °C. All of these activities were performed under sterile conditions.

#### 3.2.3. Analytical Determinations

##### Daily Weight Loss

The weight loss during fermentation, related to the release of CO_2_, was measured using a RADWAG WPS 600/C scale (Radom, Poland).

##### Ethanol Content, Real Extract and Calorific Value

The ethanol content, real extract and calorific value were measured using an automatic wort and beer analyser (Alcolyzer, Anton Paar DMA 4500+, Warsaw, Poland). A sample of degassed beer was mixed with diatomaceous earth and filtered through a paper filter to obtain ~50 mL of filtrate. The filtrate was degassed for 20 min (universal shaker, 150 rpm), adjusted to 20 °C and filtered again.

##### The pH Measurement

The pH of the beers was measured using a Mettler Toledo FiveGo (Warsaw, Poland) pH meter. The pH meter was first calibrated with respective buffer solutions.

##### Volatile Compounds

Volatile compound analysis (SPME-GC-MS): For the determination of the volatile compound contents, 1 g NaCl and a beer sample of 2 mL were added to a 10 mL vial. An internal standard solution was then added (0.57 mg/L 4-methyl-2-pentanol, 0.2 mg/L anethole and 1.48 mg/L ethyl nonanoate, Sigma-Aldrich, Saint Louis, MO, USA). A PDMS-fibre-covered SPME device was first conditioned by placing it in the injector port of a gas chromatograph at 250 °C for 1 h. To collect a sample, the fibre was introduced into the headspace, and it was mixed (300 rpm) at a temperature of 60 °C for 30 min. The SPME device was then inserted into the injector port of the Agilent Technologies 7890B chromatograph system equipped with the LECO Pegasus High-Throughput TOF-MS system and held in the inlet for 3 min. The test components were separated on an Rtx-1ms capillary column (crossbond 100% dimethylpolysiloxane, 30 m × 0.53 mm × 0.5 μm). The detector temperature was 250 °C, and the column was heated according to the following programme: 40 °C for 3 min with 8 °C increments to 230 °C, maintaining a constant temperature for 9 min. The carrier gas was helium at a constant flow rate of 1.0 mL/min. Compounds were identified using mass spectral libraries and linear retention rates calculated from the C6 to C30 series of n-alkanes. Qualitative and quantitative identification of the volatiles was based on a comparison of the retention times and peak surface areas from sample and standard chromatograms. The other components detected were determined semi-quantitatively (µg/L) by measuring the relative peak area of each identified compound according to the National Institute of Standards and Technology (NIST).

Odour-active volatile compounds in the beers were identified using olfactometry (GC-FID-O). The gas chromatograph Hewlett Packard 5890 Series II was coupled to an FID and an olfactory detector port ODP3 (Gerstel, Mülheim an der Ruhr, Germany).

The tested components were separated on a Rxi^®^-1ms capillary column (crossbond 100% dimethyl polysiloxane; 30 m × 0.53 mm × 0.5 m). Three trained GC-O analysts were asked to describe the odours they perceived.

##### Organic Acid Analysis

High-performance liquid chromatography was used to analyse the organic acids and the sugar profile. The HPLC analysis was performed using a Shimadzu (Kyoto, Japan) chromatograph equipped with a UV-Vis detector. Malic, oxalic, succinic, lactic, citric and acetic acids (Sigma-Aldrich) were determined using the Rezex ROA-Organic Acid Aminex HPX-87H (300 mm, 18 cm × 7.8 mm). Samples were eluted isocratically at 40 °C with a mobile phase (0.005 M H_2_SO_4_) at a flow rate of 0.4 mL/min [63].

##### Sugar and Glycerol Contents

The sugar content was determined using the HPLC method. The analysis of the sugar profile was carried out using a Shimadzu (Japan) NEXERA XR instrument with an RF-20A refractometric detector. The separation was performed on an Asahipak NH2P-50 4.6 × 250 mm Shodex column (Showa Denko Europe, Munich, Germany), thermostatted at 30 °C. The mobile phase consisted of an aqueous solution of acetonitrile (70%), and the isocratic elution programme (0.8 mL/min) lasted 16 min. Quantitative determinations were made using standard curves generated for the corresponding standards: glucose, maltose and glycerol. In the tables of the results, only concentrations of sugars above the detection limit of the method are shown.

##### Sensory Analysis (QDA)

The sensory evaluation was performed by ten trained testers. The aroma of the beers was characterised using the method of quantitative descriptive analysis (QDA) on a scale of 0–6 points. Awarding six points indicated that the evaluator sensed a highly intense aroma, while zero points indicated the lack of a perception of a given aroma. The intensity of the following aromas was analysed—fruity (red apple, pineapple, banana and citrus), floral (rose, geranium and honey), malty (malt and cookies), hoppy (the aroma of hops), yeasty (the aroma of yeasts) and chemical (solvent, sulfuric, faeces and pharmaceutical).

### 3.3. Statistical Analysis

The results are presented as the mean of three or more independent experiments, with bars representing the standard deviation. The data were analysed using one-way analysis of variance (ANOVA). The significance of the difference for each parameter was analysed separately using Tukey’s range test (Statistica v. 10, StatSoft Inc., Krakow, Poland).

## 4. Conclusions

A comprehensive study of selected strains for malt wort fermentation revealed the potential of *L. thermotolerans* and *L. fermentati* to produce a wide range of beers from a single wort. Analysis of the fermentation process allowed us to identify strains that showed intense fermentation and those that showed a reduced process efficiency. These results were consistent with the amount of ethanol produced. Yeast strains with a reduced fermentation efficiency were found to produce significantly less ethanol and ester compounds. In addition, the resulting beers had a higher concentration of residual extract, including maltose. Of particular interest was the yeast’s diverse ability to produce organic acids and glycerol. This analysis showed that yeast of the genus *Lachancea* can be used to produce both standard and sour beers (with a pH below 4). However, the GC-O analysis showed that sour beers and beers with a reduced alcohol content had a reduced aromatic profile compared to that of standard beer. However, it was still considered to be at an acceptable level for those kinds of beer.

## Figures and Tables

**Table 1:** Concentration of fructose, glucose, sucrose and maltose in wort and beers fermented by the strains *L. thermotolerans* and *L. fermentati*.

	Fructose	Glucose	Sucrose	Maltose	Glycerol	Real Extract
	g/L					% [*w*/*w*]
Wort	6.22 ± 1.05 ^a^	13.6 ± 2.38 ^a^	4.19 ± 1.31 ^a^	58.1 ± 2.26 ^a^	n/d	11.9 ± 0.09 ^a^
MN477031	2.52 ± 0.55 ^b^	2.56 ± 0.46 ^bf^	n/d	4.96 ± 0.34 ^b^	6.53 ± 1.31 ^a^	5.82 ± 0.04 ^bd^
PYCC7194	2.12 ± 0.27 ^bd^	1.00 ± 0.22 ^ce^	n/d	11.3 ± 1.75 ^c^	3.14 ± 0.35 ^b^	6.20 ± 0.21 ^c^
PYCC6701	4.20 ± 0.43 ^c^	2.52 ± 0.24 ^b^	2.10 ± 0.62 ^b^	17.2 ± 3.24 ^d^	6.44 ± 2.08 ^ad^	5.87 ± 0.06 ^bd^
PYCC4675	1.70 ± 0.18 ^d^	1.35 ± 0.16 ^cd^	n/d	6.18 ± 1.52 ^be^	3.1 ± 0.92 ^bc^	5.76 ± 0.02 ^b^
PYCC5883	1.54 ± 0.19 ^d^	1.86 ± 0.45 ^d^	2.77 ± 0.38 ^b^	58.1 ± 2.22 ^a^	1.59 ± 0.59 ^c^	10.6 ± 0.04 ^e^
PYCC5195	2.55 ± 0.62 ^be^	1.31 ± 0.99 ^bce^	2.50 ± 0.61 ^b^	52.5 ± 5.14 ^a^	1.80 ± 0.69 ^c^	10.1 ± 0.04 ^f^
PYCC4135	2.23 ± 0.13 ^be^	2.35 ± 0.35 ^b^	3.39 ± 0.18 ^a^	57.6 ± 1.63 ^a^	3.91 ± 1.14 ^bd^	10.3 ± 0.03 ^g^
PYCC2908	1.48 ± 0.43 ^d^	1.30 ± 0.47 ^cde^	n/d	8.18 ± 1.47 ^ce^	2.65 ± 0.79 ^bc^	5.91 ± 0.05 ^d^
PYCC6986	1.63 ± 0.82 ^bd^	0.66 ± 0.29 ^e^	n/d	4.31 ± 1.73 ^b^	3.01 ± 0.47 ^b^	5.85 ± 0.03 ^bd^
PYCC6375	1.81 ± 0.66 ^bd^	3.64 ± 0.7 ^f^	n/d	8.92 ± 2.08 ^ce^	3.16 ± 0.99 ^bc^	5.77 ± 0.02 ^b^

Notes: The results are presented as the average of three or more independent replicate experiments. The standard deviation follows the ± symbol. Values with different superscript roman letters (a–g) in the same column are significantly different according to Tukey’s range test (*p* < 0.05).

**Table 2:** Physico-chemical parameters of beers fermented by the strains *L. thermotolerans* and *L. fermentati*.

	Ethanol [% *v*/*v*]	pH	Caloric Value [kcal/100 mL]
MN477031	4.04 ± 0.06 ^af^	4.43 ± 0.04 ^a^	43.0 ± 0.37 ^abe^
PYCC7194	3.8 ± 0.08 ^b^	3.60 ± 0.13 ^b^	43.0 ± 0.35 ^abe^
PYCC6701	4.06 ± 0.03 ^a^	3.78 ± 0.07 ^b^	43.3 ± 0.05 ^a^
PYCC4675	4.03 ± 0.07 ^af^	3.94 ± 0.03 ^c^	42.7 ± 0.31 ^b^
PYCC5883	0.89 ± 0.02 ^ce^	4.65 ± 0.05 ^d^	43.5 ± 0.03 ^c^
PYCC5195	1.02 ± 0.05 ^d^	4.55 ± 0.03 ^e^	42.3 ± 0.11 ^d^
PYCC4135	0.84 ± 0.05 ^e^	4.74 ± 0.05 ^d^	42.1 ± 0.30 ^d^
PYCC2908	3.96 ± 0.03 ^f^	3.74 ± 0.03 ^b^	42.9 ± 0.27 ^abe^
PYCC6986	4.09 ± 0.06 ^af^	4.17± 0.04 ^f^	43.4 ± 0.35 ^ac^
PYCC6375	3.95 ± 0.01 ^f^	3.88 ± 0.03 ^c^	42.3 ± 0.04 ^d^

Notes: The results are presented as the average of three or more independent replicate experiments. The standard deviation follows the ± symbol. Values with different superscript roman letters (a–f) in the same column are significantly different according to Tukey’s range test (*p* < 0.05).

**Table 4:** Concentration of volatile compounds in beers fermented by the strains *L. thermotolerans* and *L. fermentati*.

Compound [µg/L]	*m*/*z*	LRI	PYCC 5883	PYCC 6701	PYCC 4675	MN 47708	PYCC 2908	PYCC 5195	PYCC 6375	PYCC 7194	PYCC 4135	PYCC 6986
2-Propanol (isopropyl alcohol)	59	480	171 ± 6.23 ^a^	116 ± 9.42 ^b^	141 ± 5.26 ^c^	59.7 ± 17.5 ^d^	160 ± 45.1 ^abcgh^	409 ± 40.2 ^e^	11.2 ± 1.32 ^f^	157 ± 4.93 ^gh^	166 ± 6.23 ^ah^	36.9 ± 17.2 ^d^
2-Methyl-3-buten-2-ol	71	611	22.8 ± 5.06 ^a^	27 ± 3.52 ^a^	4.52 ± 4.04 ^bc^	6.33 ± 0.33 ^b^	9.86 ± 3.58 ^b^	5.28 ± 1.91 ^bc^	n/d	3.34 ± 0.15 ^c^	31 ± 3.13 ^a^	n/d
2-Methyl-1-propanol (isobutyl alcohol)	33	617	150 ± 18.2 ^a^	535 ± 16.7 ^b^	558 ± 16.6 ^b^	538 ± 83.1 ^b^	373 ± 15.7 ^c^	447 ± 86.5 ^bcd^	409 ± 15.7 ^d^	435 ± 21.2 ^bd^	371 ± 9.51 ^ce^	588 ± 77.8 ^bf^
Isoamyl alcohol	42	723	1370 ± 181 ^a^	7889 ± 254 ^b^	5650 ± 422 ^c^	4318 ± 319 ^d^	5510 ± 238 ^c^	2474 ± 234	3345 ± 31.9 ^f^	2177.76 ± 160 ^e^	3171 ± 68.9 ^g^	4757 ± 262
2-Methyl-1-butanol	56	740	419 ± 41.0 ^a^	941 ± 29.2 ^b^	1157 ± 37.5 ^c^	13.6 ± 0.67 ^d^	1170 ± 75.3 ^c^	651 ± 61.2 ^e^	11.6 ± 1.00 ^f^	11.3 ± 0.73 ^f^	645 ± 5.16 ^e^	1074 ± 16.7 ^g^
L-(+)-2.3-Butanediol	29	802	n/d	18.5 ± 0.07 ^a^	n/d	n/d	n/d	n/d	n/d	n/d	n/d	19.5± 4.04 ^a^
2-Ethyl-1-hexanol	57	1016	410 ± 28.5 ^a^	242 ± 17.5 ^b^	349 ± 50.5 ^a^	233 ± 12.4 ^bc^	281 ± 63.1 ^bc^	374 ± 20.0 ^a^	201 ± 30.5 ^bce^	213 ± 7.64 ^c^	504 ± 25 ^d^	165 ± 14.8 ^e^
Dihydromyrcenol	59	1067	1.63 ± 0.31 ^abd^	1.35 ± 0.20 ^abcde^	1.09 ± 0.24 ^acef^	1.22 ± 0.19 ^acde^	1.38 ± 0.19 ^abcde^	1.57 ± 0.05 ^b^	1.18 ± 0.09 ^ce^	1.37 ± 0.02 ^d^	0.98 ± 0.25 ^ef^	0.97 ± 0.02 ^f^
Phenylethanol	91	1084	55.8 ± 22.8 ^a^	1368 ± 60.1 ^b^	1566 ± 31.1 ^c^	1720 ± 110 ^d^	1792 ± 123 ^d^	815 ± 42.8 ^e^	1323 ± 13.7 ^b^	1606 ± 78.8 ^cd^	793 ± 33.4 ^e^	1621 ± 36.1 ^cd^
4-Ethylguaiacol	137	1254	1218 ± 52 ^a^	5.04 ± 0.16 ^b^	n/d	n/d	n/d	n/d	n/d	n/d	n/d	n/d
Decanol	55	1272	n/d	n/d	2.68 ± 0.16 ^a^	3.68 ± 0.19 ^b^	n/d	n/d	n/d	n/d	n/d	n/d
Nerolidol	69	1575	n/d	2.16 ± 0.27 ^a^	2.44 ± 0.53 ^a^	1.19 ± 0.18 ^bd^	1.40 ± 0.03 ^b^	0.24 ± 0.04 ^c^	1.32 ± 0.12 ^b^	0.84 ± 0.22 ^d^	0.26 ± 0.06 ^c^	1.72 ± 0.04 ^e^
Ethyl Acetate	61	614	38.7 ± 0.6 ^a^	1544 ± 40.7 ^b^	949 ± 21.3 ^c^	937 ± 23.3 ^c^	2061 ± 327 ^d^	336 ± 19 ^e^	961 ± 6.1 ^c^	782 ± 29.7 ^f^	94.2 ± 7.6 ^g^	1147 ± 36.6 ^h^
Ethyl propanoate	57	699	1.12± 0.36 ^a^	123 ± 15.4 ^b^	61.3 ± 1.77 ^c^	45.1 ± 1.31 ^d^	101 ± 21.1 ^b^	13.4 ± 1.60 ^e^	65.3 ± 2.55 ^c^	50.9 ± 5.61 ^d^	44.4 ± 6.85 ^d^	82.3 ± 11.7 ^e^
Ethyl butanoate	71	789	1.39 ± 0.22 ^a^	6.02 ± 0.24 ^b^	2.79 ± 0.31 ^cf^	n/d	3.74 ± 0.59 ^df^	n/d	2.32 ± 0.15 ^e^	1.69 ± 0.48 ^a^	n/d	3.56 ± 0.46 ^f^
Ethyl lactate	45	806	n/d	15.4 ± 2.55 ^a^	n/d	n/d	23.1 ± 2.86 ^b^	20.3 ± 18.6 ^abc^	n/d	46.57 ± 11.96 ^c^	n/d	n/d
2-Methyl-1-butyl acetate	43	869	n/d	8.67 ± 0.14	n/d	n/d	n/d	n/d	n/d	n/d	n/d	n/d
Ethyl hexanoate	88	986	2.21 ± 0.2 ^a^	12.3 ± 0.69 ^b^	4.26 ± 0.82 ^cd^	4.97 ± 0.71 ^c^	5.99 ± 1.74 ^c^	n/d	3.60 ± 0.12 ^d^	4.31 ± 0.26 ^c^	n/d	4.09 ± 0.80 ^c^
Ethyl octanoate	88	1180	13.3 ± 3.15 ^a^	61.2 ± 6.92 ^b^	27 ± 2.40 ^c^	69.3 ± 12.5 ^b^	28.5 ± 2.73 ^c^	6.27 ± 0.19 ^d^	18.2 ± 2.59 ^af^	17.5 ± 3.03 ^af^	3.10 ± 0.23 ^e^	21.7 ± 4.30 ^cf^
2-Phenethyl acetate	104	1228	2.12 ± 0.32 ^a^	5752 ± 1991 ^b^	14.5 ± 1.50 ^c^	11.8 ± 1.98 ^cd^	15.4 ± 3.03 ^c^	8.21 ± 2.00 ^d^	8.9 ± 0.37 ^de^	13.5 ± 1.47 ^c^	5.60 ± 0.57 ^f^	6.03 ± 0.67 ^f^
Propyl nonanoate	61	1370	3.26 ± 0.16 ^a^	3.81 ± 0.58 ^a^	2.96 ± 0.33 ^ab^	2.81 ± 0.05 ^b^	2.56 ± 0.53 ^bd^	1.14 ± 0.24 ^c^	1.93 ± 0.16 ^d^	1.15 ± 0.17 ^c^	1.43 ± 0.21 ^c^	2.22 ± 0.23 ^d^
Ethyl decanoate	88	1397	0.55 ± 0.05 ^a^	7.80 ± 0.96 ^b^	9.54 ± 3.35 ^b^	2585 ± 43 ^c^	18.9 ± 4.90 ^d^	1.05 ± 0.13 ^e^	2.77 ± 0.08 ^f^	3.61 ± 0.03 ^g^	0.92 ± 0.14 ^e^	212 ± 13.5 ^h^
Ethyl dodecanoate	88	1581	3.59 ± 0.22 ^a^	6.60 ± 0.46 ^b^	3.29 ± 1.12 ^adf^	19.5 ± 3.17 ^c^	2.76 ± 0.49 ^df^	1.38 ± 0.13 ^e^	1.20 ± 0.25 ^eg^	3.15 ± 0.13 ^f^	0.98 ± 0.20 ^g^	8.48 ± 1.08 ^h^
Isoamyl acetate	43	872	44.6 ± 5.06 ^a^	250 ± 16 ^b^	68.4 ± 5.76 ^c^	70.9 ± 12.3 ^c^	74.5 ± 9.10 ^c^	36.5 ± 6.19 ^a^	59.6 ± 2.74 ^d^	79.1 ± 6.01 ^c^	15.5 ± 3.78 ^e^	112 ± 11.7 ^f^

Notes: Results are presented as the mean of three or more independent replicate experiments. The standard deviation follows the ± symbol. Values with different superscript Roman letters (a–h) in the raw column are significantly different according to Tukey’s range test (*p* < 0.05).

**Table 5:** Indication of a strain’s potential to produce a specific type of beer.

Beer Type	Recommended Strain	Odour Description	Tentative Identification (On the Basis of Odour and the MS Detector/ Suspected Compound)
Low-alcohol	PYCC4135	flower fruit tropical fruit flowers, lilies of the valley freshener ice/fresh candy flower sweet, candy candy	2-Methyl-3-buten-2-ol Isobutyl acetate Ethyl hexanoate Phenylethyl Alcohol Isoamyl alcohol Dihydromyrcenol 1-Nonanol β-Damascenone Ethyl decanoate
Sour	PYCC7194	fruit, floral boiled potatoes, sulphuric sweet, fruity, pineapple red apple roasted coffee sweet fruit mint rose floral notes	2-Methyl-3-buten-2-ol Dimethyl disulfide Ethyl lactate Ethyl hexanoate (5-Methyl-2-furyl)methanethiol ethyl isovalerate Dihydromyrcenol Phenylethyl Alcohol 1-Nonanol
Regular	MN477031	flower fruity, bubblegum cooked vegetables sweet banana fruity, pineapple fruity, banana sweet wort, berry fruity red apple roasted coffee rose oil plum	2-Methyl-3-buten-2-ol Ethyl propanoate Dimethyl disulfide Isobutyl acetate Ethyl lactate Isoamyl acetate 3-Hexen-2-one Ethyl hexanoate (5-Methyl-2-furyl)methanethiol Phenylethyl Alcohol 1-Nonanol β-Damascenone

## Data Availability

The data are contained within the article.

## References

[1] Salanță L.C., Coldea T.E., Ignat M.V., Pop C.R., Tofană M., Mudura E., Borșa A., Pasqualone A., Zhao H. (2020). Non-Alcoholic and Craft Beer Production and Challenges. Processes.

[2] Sohrabvandi S., Mortazavian A.M., Rezaei K. (2012). Health-Related Aspects of Beer: A Review. Int. J. Food Prop..

[3] Hampton A., Pham T., Du X. (2023). Impact of Flavor Factorized by Alcohol Level and Flavor Type on ‘Beer Refreshing Perception’ in a Model Study and the Exploration of Sensory Drivers for ‘Refreshing. J. Am. Soc. Brew. Chem..

[4] Krebs G., Gastl M., Becker T. (2021). Chemometric Modeling of Palate Fullness in Lager Beers. Food Chem..

[5] Schreurs M., Piampongsant S., Roncoroni M., Cool L., Herrera-Malaver B., Vanderaa C., Theßeling F.A., Kreft Ł., Botzki A., Malcorps P. (2024). Predicting and Improving Complex Beer Flavor through Machine Learning. Nat. Commun..

[6] Iorizzo M., Coppola F., Letizia F., Testa B., Sorrentino E. (2021). Role of Yeasts in the Brewing Process: Tradition and Innovation. Processes.

[7] Hejna A. (2022). More than Just a Beer—The Potential Applications of by-Products from Beer Manufacturing in Polymer Technology. Emergent. Mater..

[8] Lei H., Zhao H., Yu Z., Zhao M. (2012). Effects of Wort Gravity and Nitrogen Level on Fermentation Performance of Brewer’s Yeast and the Formation of Flavor Volatiles. Appl. Biochem. Biotechnol..

[9] Postigo V., Esteban S., Arroyo T. (2023). Lachancea Thermotolerans, an Innovative Alternative for Sour Beer Production. Beverages.

[10] Langstaff S.A., Lewis M.J. (1993). THE MOUTHFEEL OF BEER—A REVIEW. J. Inst. Brew..

[11] Carlquist M., Gibson B., Karagul Yuceer Y., Paraskevopoulou A., Sandell M., Angelov A.I., Gotcheva V., Angelov A.D., Etschmann M., de Billerbeck G.M. (2015). Process Engineering for Bioflavour Production with Metabolically Active Yeasts—A Mini-Review. Yeast.

[12] Missbach B., Majchrzak D., Sulzner R., Wansink B., Reichel M., Koenig J. (2017). Exploring the Flavor Life Cycle of Beers with Varying Alcohol Content. Food Sci. Nutr..

[13] Olšovská J., Štěrba K., Vrzal T., Čejka P. (2019). Nutritional Composition and Energy Value of Different Types of Beer and Cider. Kvasny Prumysl..

[14] Porretta S., Donadini G. (2008). A Preference Study for No Alcohol Beer in Italy Using Quantitative Concept Analysis. J. Inst. Brew..

[15] Cubillos F.A., Gibson B., Grijalva-Vallejos N., Krogerus K., Nikulin J. (2019). Bioprospecting for Brewers: Exploiting Natural Diversity for Naturally Diverse Beers. Yeast.

[16] Zdaniewicz M., Satora P., Pater A., Bogacz S. (2020). Low Lactic Acid-Producing Strain of Lachancea Thermotolerans as a New Starter for Beer Production. Biomolecules.

[17] Larroque M.N., Carrau F., Fariña L., Boido E., Dellacassa E., Medina K. (2021). Effect of Saccharomyces and Non-Saccharomyces Native Yeasts on Beer Aroma Compounds. Int. J. Food Microbiol..

[18] Holt S., Mukherjee V., Lievens B., Verstrepen K.J., Thevelein J.M. (2018). Bioflavoring by Non-Conventional Yeasts in Sequential Beer Fermentations. Food Microbiol..

[19] Li R., Sun Y. (2019). Effects of Honey Variety and Non-Saccharomyces Cerevisiae on the Flavor Volatiles of Mead. J. Am. Soc. Brew. Chem..

[20] Gatto V., Binati R.L., Lemos Junior W.J.F., Basile A., Treu L., de Almeida O.G.G., Innocente G., Campanaro S., Torriani S. (2020). New Insights into the Variability of Lactic Acid Production in Lachancea Thermotolerans at the Phenotypic and Genomic Level. Microbiol. Res..

[21] Morata A., Loira I., Tesfaye W., Bañuelos M.A., González C., Suárez Lepe J.A. (2018). Lachancea Thermotolerans Applications in Wine Technology. Fermentation.

[22] Vicente J., Navascués E., Calderón F., Santos A., Marquina D., Benito S., Fracassetti D., Rustioni L. (2021). An Integrative View of the Role of Lachancea Thermotolerans in Wine Technology. Foods.

[23] Madden A.A., Lahue C., Gordy C.L., Little J.L., Nichols L.M., Calvert M.D., Dunn R.R., Smukowski Heil C. (2022). Sugar-Seeking Insects as a Source of Diverse Bread-Making Yeasts with Enhanced Attributes. Yeast.

[24] Siesto G., Pietrafesa R., Tufariello M., Gerardi C., Grieco F., Capece A. (2023). Application of Microbial Cross-over for the Production of Italian Grape Ale (IGA), a Fruit Beer Obtained by Grape Must Addition. Food Biosci..

[25] Vicente J., Vladic L., Navascués E., Brezina S., Santos A., Calderón F., Tesfaye W., Marquina D., Rauhut D., Benito S. (2024). A Comparative Study of Lachancea Thermotolerans Fermentative Performance under Standardized Wine Production Conditions. Food Chem. X.

[26] Aredes R.S., Peixoto F.C., Sphaier L.A., Silva V.N.H., Duarte L.M., de Carvalho Marques F.F. (2023). Determination of Carbohydrates in Brewer’s Wort by Capillary Electrophoresis with Indirect UV Detection. J. Food Compos. Anal..

[27] D’Amore T., Russell I., Stewart G.G. (1989). Sugar Utilization by Yeast during Fermentation. J. Ind. Microbiol..

[28] Fairbairn S., Engelbrecht L., Setati M.E., du Toit M., Bauer F.F., Divol B., Rossouw D. (2021). Combinatorial Analysis of Population Dynamics, Metabolite Levels and Malolactic Fermentation in Saccharomyces Cerevisiae/ Lachancea Thermotolerans Mixed Fermentations. Food Microbiol..

[29] du Plessis H.W., du Toit M., Hoff J.W., Hart R.S., Ndimba B.K., Jolly N.P. (2017). Characterisation of Non-Saccharomyces Yeasts Using Different Methodologies and Evaluation of Their Compatibility with Malolactic Fermentation. South Afr. J. Enol. Vitic..

[30] Sroka P., Tuszyński T. (2007). Changes in Organic Acid Contents during Mead Wort Fermentation. Food Chem..

[31] Zdaniewicz M., Poreda A., Tuszyński T. (2016). Rotary Jet Head—A Device for Accelerating the Fermentation Process in Brewing. J. Inst. Brew..

[32] Sampaolesi S., Pérez-Través L., Pérez D., Roldán López D., Briand L.E., Pérez-Torrado R., Querol A. (2023). Identification and Assessment of Non-Conventional Yeasts in Mixed Fermentations for Brewing Bioflavored Beer. Int. J. Food Microbiol..

[33] Shimizu H., Mizuno S., Hiroshima T., Shioya S. (2002). Effect of Carbon and Nitrogen Additions on Consumption Activity of Apparent Extract of Yeast Cells in a Brewing Process. J. Am. Soc. Brew. Chem..

[34] Magalhães F., Vidgren V., Ruohonen L., Gibson B. (2016). Maltose and Maltotriose Utilisation by Group I Strains of the Hybrid Lager Yeast Saccharomyces Pastorianus. FEMS Yeast Res.

[35] Toh D.W.K., Chua J.Y., Lu Y., Liu S.Q. (2020). Evaluation of the Potential of Commercial Non-Saccharomyces Yeast Strains of Torulaspora Delbrueckii and Lachancea Thermotolerans in Beer Fermentation. Int. J. Food Sci. Technol..

[36] Galaz V., Franco W. (2023). Lachancea Quebecensis a Novel Isolate for the Production of Craft Beer. Foods.

[37] Cioch-Skoneczny M., Krystian K., Satora P., Skoneczny S., Zdaniewicz M., Pater A. (2020). The Use of Non-Saccharomyces Yeast and Enzymes in Beer Production. Acta Univ. Cibiniensis-Ser. E Food Technol..

[38] Kunze W., Hendel O. (2019). Technology Brewing and Malting.

[39] Domizio P., House J.F., Joseph C.M.L., Bisson L.F., Bamforth C.W. (2016). Lachancea Thermotolerans as an Alternative Yeast for the Production of Beer. J. Inst. Brew..

[40] Monošík R., Magdolen P., Streďanský M., Šturdík E. (2013). Monitoring of Monosaccharides, Oligosaccharides, Ethanol and Glycerol during Wort Fermentation by Biosensors, HPLC and Spectrophotometry. Food Chem..

[41] Zhao X., Procopio S., Becker T. (2015). Flavor Impacts of Glycerol in the Processing of Yeast Fermented Beverages: A Review. J. Food Sci. Technol..

[42] Sohrabvandi S., Mousavi S.M., Razavi S.H., Mortazavian A.M., Rezaei K. (2010). Alcohol-Free Beer: Methods of Production, Sensorial Defects, and Healthful Effects. Food Rev. Int..

[43] Zdaniewicz M., Pater A., Knapik A., Duliński R. (2021). The Effect of Different Oat (*Avena Sativa* L) Malt Contents in a Top-Fermented Beer Recipe on the Brewing Process Performance and Product Quality. J. Cereal Sci..

[44] Osburn K., Amaral J., Metcalf S.R., Nickens D.M., Rogers C.M., Sausen C., Caputo R., Miller J., Li H., Tennessen J.M. (2018). Primary Souring: A Novel Bacteria-Free Method for Sour Beer Production. Food Microbiol..

[45] Baroň M., Fiala J. (2012). Chasing after Minerality, Relationship to Yeast Nutritional Stress and Succinic Acid Production. Czech J. Food Sci..

[46] Tyrell T. (2014). Strategies for Reducing Succinic Acid Concentrations in Beer. J. Am. Soc. Brew. Chem..

[47] Li H., Liu F. (2015). Changes in Organic Acids during Beer Fermentation. J. Am. Soc. Brew. Chem..

[48] Olaniran A.O., Hiralal L., Mokoena M.P., Pillay B. (2017). Flavour-Active Volatile Compounds in Beer: Production, Regulation and Control. J. Inst. Brew..

[49] Kobayashi M., Nagahisa K., Shimizu H., Shioya S. (2006). Simultaneous Control of Apparent Extract and Volatile Compounds Concentrations in Low-Malt Beer Fermentation. Appl. Microbiol. Biotechnol..

[50] Peddie H.A.B. (1990). Ester Formation in Brewery Fermentations. J. Inst. Brew..

[51] Williamson S.A., Iverson W.G. (1993). Determination of Short-Chain Diols and Selected Fermentation By-Products in Beer. J. Am. Soc. Brew. Chem..

[52] Olsen A., Christensen B.W., Madsen J.I. (1988). Onion-Like Off-Flavour in Beer: Isolation and Identification of The Culprits. Carlsberg Res. Commun..

[53] Saerens S.M.G., Delvaux F., Verstrepen K.J., Van Dijck P., Thevelein J.M., Delvaux F.R. (2008). Parameters Affecting Ethyl Ester Production by Saccharomyces Cerevisiae during Fermentation. Appl. Environ. Microbiol..

[54] Strong G., England K. (2021). Beer Judge Certification Program 2021 Beer Style Guidelines.

[55] Lermusieau G., Bulens M., Collin S. (2001). Use of GC-Olfactometry to Identify the Hop Aromatic Compounds in Beer. J. Agric. Food Chem..

[56] Wilson A., Johnson J.B., Batley R., Lal P., Wakeling L., Naiker M. (2021). Authentication Using Volatile Composition: A Proof-of-Concept Study on the Volatile Profiles of Fourteen Queensland Ciders. Beverages.

[57] Piry J., Prlhla A., Ďurčanská J., Farkaš P. (1995). Fractionation of Volatiles from Blackcurrant (*Ribes nigrum* L.) by Different Extractive Methods. Food Chem..

[58] Alegre Y., Sáenz-Navajas M.P., Ferreira V., García D., Razquin I., Hernández-Orte P. (2017). Rapid Strategies for the Determination of Sensory and Chemical Differences between a Wealth of Similar Wines. Eur. Food Res. Technol..

[59] Langos D., Granvogl M., Schieberle P. (2013). Characterization of the Key Aroma Compounds in Two Bavarian Wheat Beers by Means of the Sensomics Approach. J. Agric. Food Chem..

[60] Fritsch H.T., Schieberle P. (2005). Identification Based on Quantitative Measurements and Aroma Recombination of the Character Impact Odorants in a Bavarian Pilsner-Type Beer. J. Agric. Food Chem..

[61] Schieberle P. (1991). Primary Odorants of Pale Lager Beer Differences to Other Beers and Changes during Storage. Z. Lebensm. Unters. Forch..

[62] Tokita K., Takazumi K., Oshima T., Shigyo T. (2014). A New Method for Analyzing the Characteristic Flavor of Beer Using Selectable One-Dimensional or Two-Dimensional Gas Chromatography-Olfactometry/ Mass Spectrometry. J. Am. Soc. Brew. Chem..

[63] Satora P., Semik-Szczurak D., Tarko T., Bułdys A. (2018). Influence of Selected Saccharomyces and Schizosaccharomyces Strains and Their Mixed Cultures on Chemical Composition of Apple Wines. J. Food Sci..

